# Differential Connectivity in Colorectal Cancer Gene Expression Network

**DOI:** 10.29252/.23.1.34

**Published:** 2019-01

**Authors:** Fereshteh Izadi

**Affiliations:** Sari Agricultural Sciences and Natural Resources University (SANRU), Farah Abad Road, Mazandaran 4818168984, Iran

**Keywords:** Colon cancer, Colorectal cancer, Transcriptional networks

## Abstract

**Background:**

Colorectal cancer (CRC) is one of the challenging types of cancers; thus, exploring effective biomarkers related to colorectal could lead to significant progresses toward the treatment of this disease.

**Methods:**

In the present study, CRC gene expression datasets have been reanalyzed. Mutual differentially expressed genes across 294 normal mucosa and adjacent tumoral samples were then utilized in order to build two independent transcriptional regulatory networks. By analyzing the networks topologically, genes with differential global connectivity related to cancer state were determined for which the potential transcriptional regulators including transcription factors were identified.

**Results:**

The majority of differentially connected genes (DCGs) were up-regulated in colorectal transcriptome experiments. Moreover, a number of these genes have been experimentally validated as cancer or CRC-associated genes. The DCGs, including *GART*, *TGFB1*, *ITGA2*, *SLC16A5*, *SOX9*, and *MMP7*, were investigated across 12 cancer types. Functional enrichment analysis followed by detailed data mining exhibited that these candidate genes could be related to CRC by mediating in metastatic cascade in addition to shared pathways with 12 cancer types by triggering the inflammatory events.

**Conclusion:**

Our study uncovered correlated alterations in gene expression related to CRC susceptibility and progression that the potent candidate biomarkers could provide a link to disease.

## INTRODUCTION

Colorectal cancer (CRC) is a fatal malignancy with estimated 1.4 million cases yearly[1]. In spite of conducting leading researches to elucidate the molecular processes that advocate the normal colorectal cells toward cancer, the rate and average years of survival have not profoundly changed over decades. Experimental evidence has demonstrated the function of a certain number of genes such as *HMGA1*, *TACSTD2*[2], *SLC6A4*[3], *COL3A1*[4], *ITGA2*[5], *TXNDC17*[6], and *PPP2R5A*[7] in CRC. Although the association of genes in CRC has been presented in a number of research works[8,9], employing robust algorithms in network mining and topology analysis offers an unprecedented opportunity in depicting the etiology of cancers. Rewiring of the biological networks, to detect differentially co-regulated (DRGs) and co-expressed genes (DCG), could simplify the network’s components observation and assist to depict the relationships between interconnected genes. Gene co-expression networks enable to highlight molecular mechanisms underlying diseases[10] and can be accounted as an efficient way to assess CRC. Generally, tools designed for recovering gene regulatory interactions rely on similarity matrices indirectly measured by correlation matrices or mutual information. These matrices usually include many indirect links that should be identified and removed for increasing the reliability of gene regulatory network (GRN) inference algorithms. Hence, several sophisticated approaches have attempted to remove indirect interactions and to detect the causal relationships between gene pairs. Differential co-expression analysis aids to detect gene with different connectivity in the disease state and offers a powerful approach for elucidating transcriptome patterns and dysfunction of gene expression underlying phenotypic changes[11]. A plenty number of differentially co-expression network methods have been proposed in the literature[12-17]. For instance, DCGs and links (DCGL) attempts to identify DRGs and its links (DRLs) by comparing the expression datasets of disease and normal states[11,12]. Weighted gene co-expression network analysis (WGCNA) is a relatively new statistical method not only infers correlation patterns between two genes but also covers neighborhoods across expression data[13].

In this work, instead of DRGs, we focused on genes with differential connectivity in cancer state versus normal condition. These genes, indicating hubs within the network, supposedly to be key units controlling a wide range of essential cellular functions in a specific process like cancers. Thus, the presence of potential differential interactions through CRC genes expression datasets has been investigated. We mainly aimed to uncover the mediated relationships between genes using *in silico* approaches. The differentially expressed connected genes and molecular pathways, which we previously thought to influence the pathogenesis of CRC, were subsequently prioritized.

## MATERIALS AND METHODS

### Used datasets and pre-processing

In this work, we collected samples of normal human colorectal mucosa and adjacent CRC of four independent whole genome expression series (single-color Affymetrix Human Genome U133 Plus 2.0 Array). After a comprehensive search in NCBI Gene Expression Omnibus (GEO) (https://www.ncbi.nlm. nih.gov/gds/?term=), series with accession numbers GSE4183[18], GSE8671[19], GSE9348[20], and GSE18105[21] consisting of 84 normal and 210 CRC cases were collected. Raw CEL files of these samples, based on the platform of GPL6244, were normalized with robust multi-array average (RMA) expression measure[22] method by using the linear models for microarray data (LIMMA) R package[23] (R software v. 3.2.5). After removing ambiguous probes, the extracted probe IDs were transformed into 21654 unique and validated official gene symbols. After normalization, differentially expressed genes (DEGs) were identified between cancer and normal mucosa if the expression level alteration was above the defined threshold (fold-change >2.0 or <0.5 and adjusted *p* value <0.01) by employing LIMMA R package. The defined threshold prevents withdrawing genes with lower differential alteration.

### Assessing regulatory interactions and topological analysis

The expression values of mutual DEGs among the GSE4183, GSE8671, GSE9348, and GSE18105 series were used to construct two independent GRNs, one from 84 normal samples and another from 210 CRC samples, by employing Graphical Gaussian Models (GGM), as implemented in GeneNet R package[24]. To cover all of the mutual DEGs between control and CRC samples and more sparsity, only the 1500 top ranking edges were visualized in Cytoscape (v. 3.4.0). Using Cytoscape’s built-in Network Analyzer, we set the nodes with higher degrees and betweenness with a darker shade and bigger size, respectively. In order to analyze the topology of two independent constructed GRNs, betweenness centrality (the percentage of times a node appears on the shortest path between all pairs of nodes in the network), as a network centrality parameter, was calculated. Genes with higher betweenness centrality score, as globally connected genes, were then identified, through the CytoNCA[25]. Globally connected nodes were determined if they were in the top 40% of betweenness centrality score distribution; genes ranked between top 1–40%. Based on Yu *et al*.’s[26] report, the selection of genes in the range of 10-40% of this distribution did not have a significant effect on the results. These genes thereof were remarked as differential connected genes (DCGs) between normal and CRC conditions. Afterward, in order to extract transcription factor (TF)-gene regulatory interactions among the DCGs, a list of 9905 regulatory links between human gene TF was obtained from TRRUST database[27]. These regulatory connections were collected from 11,237 over 20 million PubMed articles and experimentally validated transcriptional regulations consisting of 821 human TFs and 2,159 target genes of TFs.

### Pathway, gene ontology (GO) enrichment, and expression pattern analysis

Mutual DEGs were separately classified by utilizing KEGG[28] to underlying pathways and to GO molecular labels by Enrichr[29]. DCGs were finally fed into pathwAX web server[30] to find a network crosstalk of significant pathways. PathwAX contains KEGG pathway information in addition to networks of gene-gene links in model organisms. The expression patterns of DCGs was ultimately sought by GEMMA database[31]. To examine the extent to which the DCGs are CRC specific in contrast to being generally expressed in several cancer types, we obtained DEGs from bladder, breast, colon, esophagus, kidney, liver, lung, skin, ovary, prostate, sarcoma, and thyroid cancers with accession numbers GSE11545[32], GSE20437[33], GSE25071[34], GSE34619[35], GSE2 0602[36], GSE49515[37], GSE43346[38], GSE6887[39], GSE14407[40], GSE45016[41], GSE2719[42], and GSE53072[43], respectively. As described before, the threshold cut-off of fold-change >2.0 or <0.5 and adjusted *p* value < 0.01 was employed to extract DEGs in 12 cancer types. The power analysis of the selected genes was conducted by calculating the survival time statistics based on the log-rank test and visualized as Kaplan-Meier survival curve[44].

## RESULTS

### Differential expression of CRC-related genes across healthy mucosa and adjacent tumor tissues

**Fig. 1:**
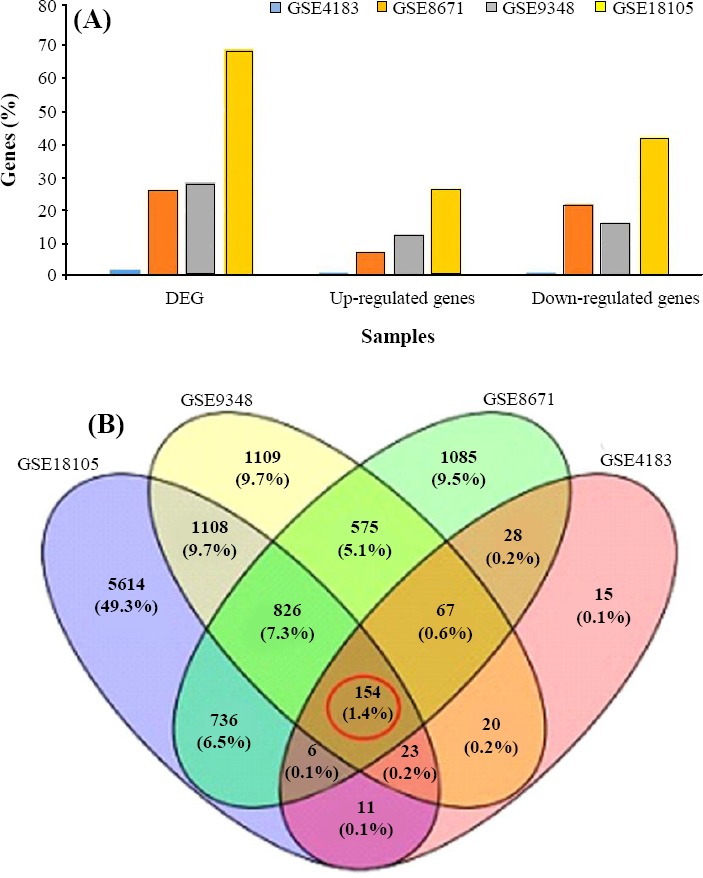
Differentially expressed genes (DEGs) in colorectal cancer-associated datasets. (A) The distribution of DEGs as well as up- and down-regulated genes in four used CRC experiments. The bars have been arranged to illustrate the percent of genes assigned to each experiment. (B) Venn diagram of intersection among the expression values of DEGs across GSE4183, GSE8671, GSE9348, and GSE18105 series (absolute log fold-change >1, absolute log fold-change <0.5, and adjusted *p* value <0.01). Ultimately, 154 genes were taken as mutual DEGs among these datasets.

Our major criteria for selecting the four aforementioned expression arrays was the avoidance of pooling transcriptome data of cell lines, *in vitro* assays, or gene expression measurement under any treatment. Genes whose expression level in normal colon mucosa displayed 2> or < 0.5 fold-change at adjusted *p* value <0.01, in comparison to tumour tissues, were selected as statistically significant DEGs. The range of DEGs through these series was unequal from 392 genes in GSE4183 to 11591 in GSE18105 (Fig. 1A). In addition, among the expression values of DEGs among

GSE4183, GSE8671, GSE9348, and GSE18105 series, 154 genes were identified as mutual DEGs (Fig. 1B).

**Fig. 2:**
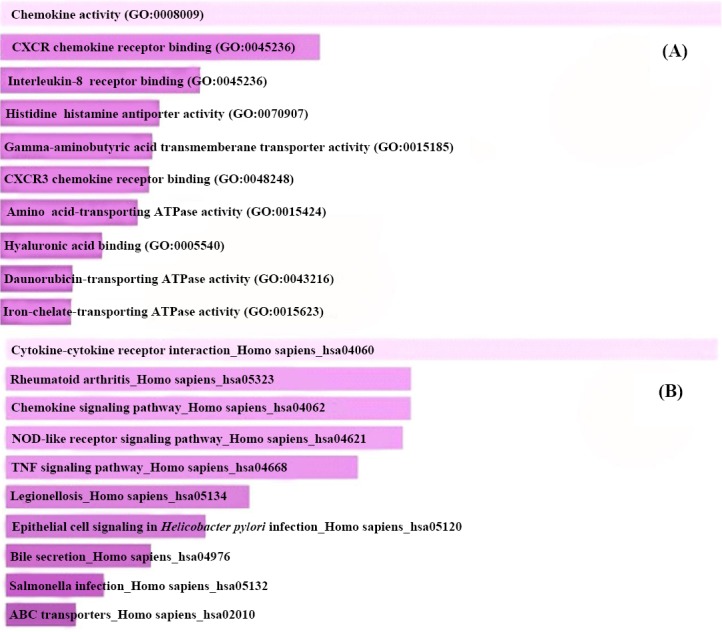
Enrichment analysis of differentially expressed genes (DEGs) in colorectal cancer datasets. (A) Functional classification of biological processes; (B) biological pathways in which mutual DEGs are involved by Enrichr and KEGG databases, respectively with default setting. The bars have been arranged top to down illustrating the number of DCGs, and significance level assigned to each GO molecular terminology and biological pathway.

As shown in Fig. 2A, Fig. 2 gene products of mutual DEGs were enriched in significantly over-represented GO molecular relevance, including cytokine and chemokine processes, in addition to tumor necrosis factor (TNF), oligomerization domain (NOD), and epithelial cell signaling pathways (Fig. 2B).

### Networking of CRC-associated datasets

**Fig. 3:**
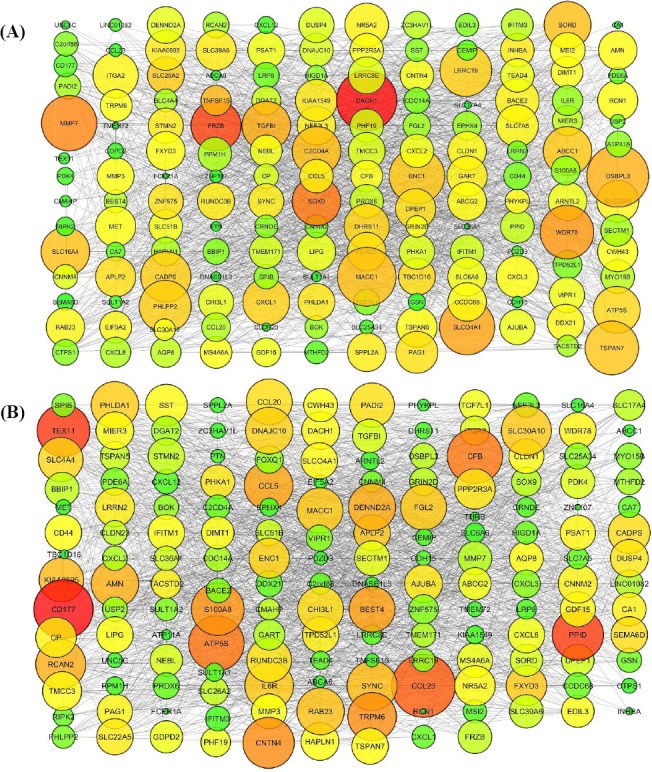
Reconstructing two independent differential regulatory networks using normal colorectal mucosa and adjacent tumor tissues, by utilizing the expression values of mutual differentially expressed genes (DEGs) across datasets. (A) Cancer and (B) healthy transcriptional regulatory networks derived by GGM algorithm. Using NetworkAnalyzer Cytoscape plug-in, degree and betweenness parameters have been mapped to node size and color so that darker and bigger nodes show higher degree and betweenness centrality.

Here, we employed R implementation of GGM as GRN inference algorithm to recognize and remove indirect links between shared DEGs. To this end, we reconstructed two independent GRNs, from 84 normal and 210 cancer cases, but both of GRNs were composed of the same expression values of 154 mutual DEGs across healthy and diseased conditions. To cover all the 154 nodes and more sparsity, we only selected the 1500 highly ranked edges between mutual DEGs (Fig. 3). In order to identify genes with differential connectivity in CRC, by exploiting CytoNCA Cytoscape plugin, 40% of the top globally connected genes (a number of shortest paths with other nodes namely betweenness) from CRC and normal networks were selected separately. Betweenness characteristic, as a centrality measurement, indicates how significant a node would be in healthy and diseased GRNs. Identifying the central nodes by these measures seemingly provides genes that modulate responses to various cellular conditions. From 154 mutual DEGs in each of CRC and healthy GRNs, 61 genes (40%) were selected based on their importance in the network by calculating the betweenness as centrality parameter. From these 61 genes, 21 genes indicated an overlap in two GRNs that were removed from the analysis as we strictly wanted to evaluate the genes that are ranked in the network by connection type variations. Ultimately, 40 genes with more relevance to CRC were selected for further analysis as DCGs.

### Filtering the CRC-related candidates across experts-curated databases

**Fig. 4:**
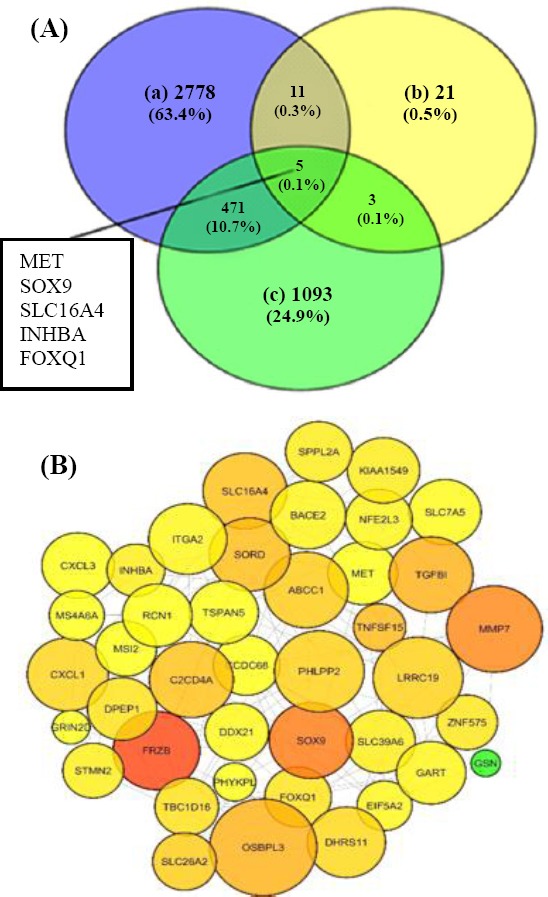
Characterizing a network of differentially connected genes (DCGs) along with topology feature analysis. (A) Venn diagrams of a, 3265 gene from DisGeNET database; b, DCGs; c, 1572 genes from Network of Cancer Genes (NCG); five genes were taken mutual colorectal cancer-associated genes. (B) Regulatory interactions of DCGs, using NetworkAnalyzer Cytoscape plug-in, degree and betweenness parameters have been mapped to node size and color so that darker and bigger nodes show higher degree and betweenness centrality.

**Fig. 5:**
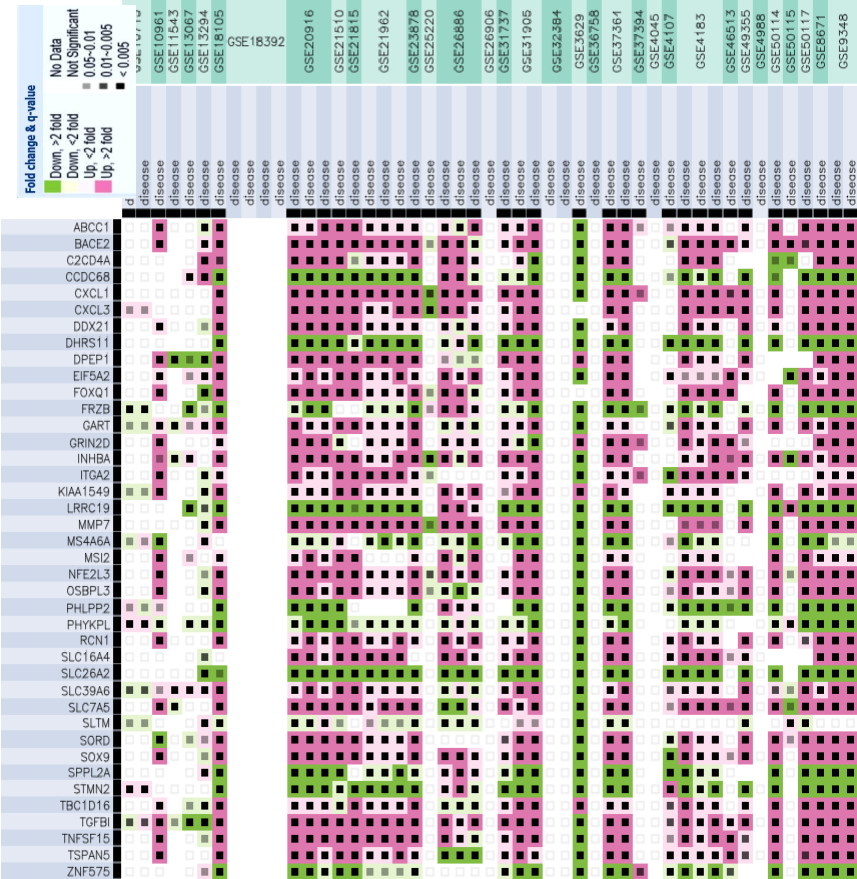
Expression profile of differentially connected genes (DCGs) across 32 colorectal cancer transcriptome datasets by GEMMA database. Dark purple, light purple, dark green, and light green show up-regulation >2 at *p* value 0.005, up-regulation >2 at *p* value 0.01-0.005, down-regulation < 2 at *p* value 0.005, down-regulation >2 at *p* value 0.01-0.005, respectively.

For the identification of CRC-related candidates among the DCGs, we intersected the DCGs with two lists of genes: 1572 genes from Network of Cancer Genes (NCG) database[45] and 3265 CRC associated genes from DisGeNET v. 2.0 database[46]. Consequently, *INHBA*, *FOXQ1*, *MET*, *SLC16A4*, *SOX9*, and *MET* were found to be common genes (Fig. 4A). DisGeNET contains a list of diseases-associated genes collection based on the presence of genetic overlaps between diseases collected from UNIPROT, human CTD, PsyGeNET, Orphanet, and the HPO. From this collection, only genes with at least one evidence from Pfam 31.0 were selected for intersection. Lower coverage of DCGs with NCG genes (17.5%) fuels the efforts in the validation of DCGs associations with CRC by experimentalists. The expression pattern of DCGs was finally checked in 33 transcriptomic experiments via GEMMA database. The expression pattern analysis through several CRC transcriptome datasets exhibited the up-regulation of the majority of DCGs. Expect for *ZNF575*, *STMN2*, *SPPL2A*, *SLC26A2*, *PHYKPL*, *PHLPP2*, *LRRC19*, *FRZB*, *DHRS1*, and *CCDC68* with a relative down-regulation (fold-change <2 at *p* values 0.01-0.005), the rest of DCGs indicated up-regulation (fold-change >2 at *p* value 0.01-0.005), as indicated in Figure 5.

### Identifying biological regulators of differentially connected genes in CRC

**Fig. 6:**
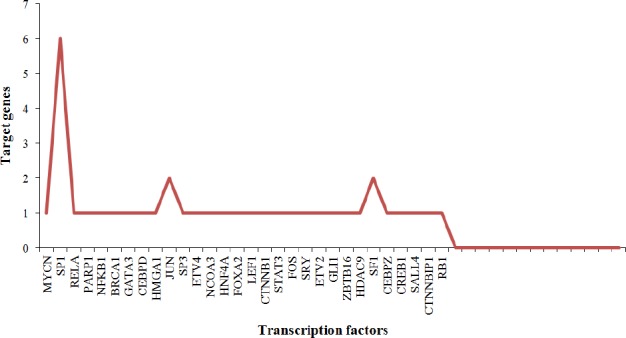
Potential regulators of differentially connected genes (DCGs). The distribution of transcription factors that modulate the expression of DCGs based on TRRUST database.

The complex molecular interactions underlying cancer genesis warrants the identification of biological entities viz. Therefore, inferring regulatory links between TFs, as transcriptional regulators, promisingly will reveal interesting aspects of DCGs. In the next step, we sought potential TFs associated with a circuits of 40 arbitrary DCGs obtained by TRRUST database. Among the DCGs, ABCC1, BACE2, CXCL1, DDX21, ITGA2, MMP7, SLC7A5, SOX9, STMN2, and TGFBI were found to be regulated by 30 TFs, mainly in an activating way. Except for the CXCL1, MMP7, and SOX9 that were regulated by 8, 9, and 10 TFs, respectively (SP1 TF was the common regulator of MMP7 and SOX9), the rest of DCGs were being regulated by distinct TFs. However, SP1, JUN, and SF1 TFs regulated more than one DCG, and the rest of TFs acted as a regulator of just one DCG (Fig. 6).

### Expression pattern of selected genes across different cancer types

**Fig. 7:**
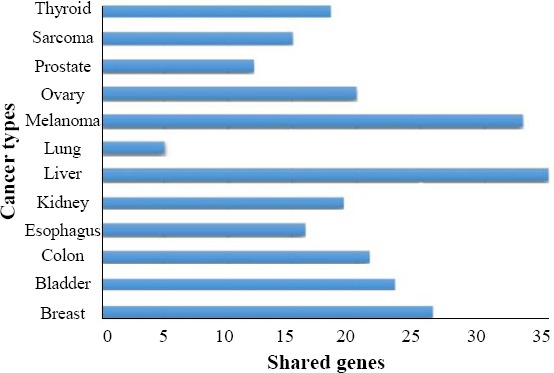
The expression pattern of differentially connected genes (DCGs) within the statistically significant differentially expressed genes (DEGs) across different cancer types (absolute log fold-change >1, absolute log fold-change <0.5, and adjusted *p* <0.01). The bars have been arranged to illustrate the number of genes shared between DCGs and each cancer type.

**Fig. 8:**
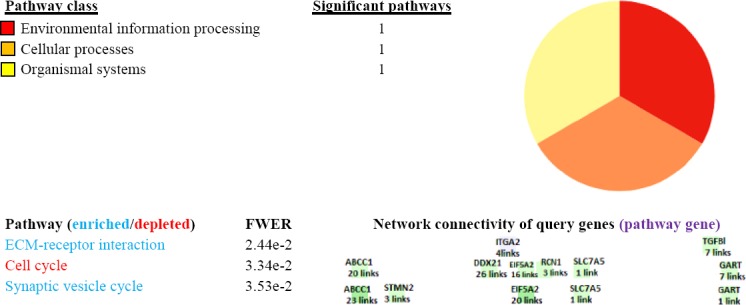
PathwAX results for the differentially connected genes (DCGs) in the colorectal cancer datasets. The Table and Pie chart summarize the pathway distribution. The Table shows enriched (blue) and depleted (red) pathways at *q* value of 0.05 as defined cut-off threshold. Darker shades in colored boxes within the table indicate higher connectivity (links) that a query gene has.

DCGs were compared to statistically significant DEGs from 12 cancer types (bladder, breast, colon, esophagus, kidney, liver, lung, skin, ovary, prostate, sarcoma, and thyroid cancers) to identify the extent to which DCGs are CRC specific (supplementary S 13-1, 13-12). It would be a strong support if DEGs identified in this study are specific to CRC, then it shows that the computational methods have likely discovered CRC-associated biomarkers correctly. For this purpose, we took the intersection of DEGs obtained from each cancer type and DCGs separately enriched the shared genes to biological pathways (data not shown). Liver and skin (>80%) as well as lung, prostate, sarcoma, and esophagus cancers (<20%) shared the most and the least genes with DCGs, respectively (Fig. 7). The shared genes principally enriched in cytokine and TNF signaling (bladder, breast, colon, kidney, liver, melanoma, and sarcoma), epithelial cell signaling, carbon metabolism (esophagus, lung, prostate, and thyroid), and PI3K-Akt signaling pathway (ovary). As the most cancers shared a few number of genes with DCGs, the finest explanation could be the roughly specific roles that the DCGs play as central nodes in CRC by mediating in PathwAX-derived pathways like extracellular matrix (ECM)-receptor interaction and synaptic vesicle cycle (Fig. 8). In sum, *CXCL3*, *SLC7A5*, *SLC26A2*, *GART*, and *CCDC68* genes were differentially expressed in three or more cancer types (Table 1).

**Table 1:** The expression pattern of DCGs across different cancer types

Shared gene	Cancer type	Expression pattern	Fold- change
*CXCL3*	Breast	Up	< 2
*SLC7A5*	Bladder	Up	< 2
*SLC26A2*	Sarcoma Thyroid	Down	>2
*GART*	Prostate	Up	< 2
	Ovary		
	Melanoma Lung		
*CCDC68*	Liver	Down	>2
	Kidney		
	Esophagus Colon		

Up, upregulation; Down, downregulation

### Validating the differentially connected genes by power analysis

**Fig. 9:**
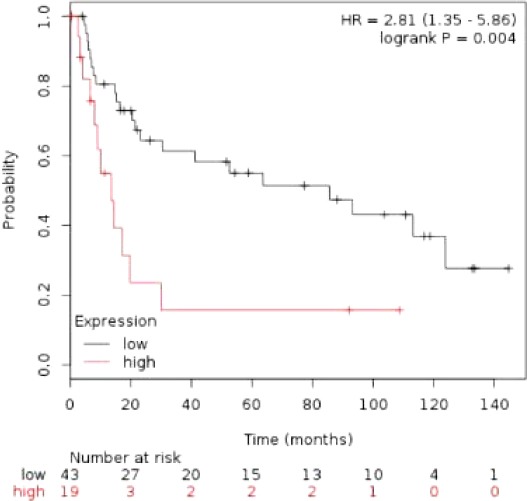
Power analysis of differentially connected genes (DCGs) in colorectal cancer datasets and Kaplan-Meier analysis of colorectal cancer dataset using the DCGs. The *p* values are computed using log-rank.

Since having been differentially expressed in cancer state in comparison to normal tissues, 14 DCGs can represent potential genes for CRC prognosis. We therefore checked the importance of 14 DCGs in CRC progression by plotting Kaplan-Meier survival curves. The survival curves was plotted by feeding DCGs in Kaplan Meier-plotter. As a result, the DCGs were predictive of CRC at *p* value = 0.004 with hazard ratio of 2.81 (Fig. 9).

## DISCUSSION

By capturing the molecular roadmap underlying human diseases, systems biology paves the path to better understanding of diseases mechanism, biomarker identification and drug discovery. CRC is still the major causes of cancer death worldwide; hence, discovering a system of biomarkers triggering the initiation and progression of CRC is a challenging topic in cancer biology[47]. The identification of co-expressed genes related to cancers presumably provides new insights in networks underlying cancers. In other words, a combination of gene effects likely holds promise as a more effective approach for detecting disease-associated genes[48]. In fact, examining co-expressed genes in contrast to the individual genes, could be more informative to explore new biomarkers[49,50]. Hereby, varied correlation between two genes in distinct states such as healthy and diseased conditions is recovered as differential co-expression. As the correlation between two genes may alter free from the expression levels of two genes, transcriptome analysis exclusively based on the differential transcript profiling impedes the structured description of regulatory patterns[51]. Several studies have therefore conducted differential co-expression analysis to facilitate the deconvolution of cellular networks in cancers[52,53]. In the present work, freely available data sources and bioinformatics tools have been exploited to infer DRGs whose interactions hypothetically promote colorectal cancer. Furthermore, attempt was made to disclose the likely relevant molecular pathways implicated by central nodes through a network of these genes. To increase the statistical power, we performed a meta-analysis, combined of multiple Affymetrix experiments. On the other hand, to decrease the experimental specific batch effects, each experiment was processed independently. Of note, we focused on the colon mucosa samples from which nearly all CRC starts. Consequently, 154 statistically significant mutual DEGs across the healthy mucosa and adjacent tumor tissues were extracted that overrepresented with chemokine and cytokine processes, signaling pathways, and transporters. In this context, we reconstructed two independent GRNs by employing GGM algorithm, using the expression values of 154 mutual DEGs. GGM algorithm produces a high-fidelity representation of the cellular network topology as a graph by recognizing regulatory interactions from non-regulatory interactions and removing non-causal links. Indeed, the casual impact of a TF on its target genes is being inferred. The critical idea behind this algorithms is the modeling of partial correlation as a measure of independence of any two genes. The assumption of inferring gene network using GGM algorithm is that the selected 154 genes are in the same pathway (or network), and they are interacting and regulating each other. However, based on the definition of DEG, these 154 genes are not necessarily related to each other. The expression correlation observed may be due to indirect regulation. In this context, the betweenness analysis will highlight genes that being regulated by the most number of other genes, and those genes with least number of connections are true important regulators. Then two inferred GRNs were topologically analyzed to find DCGs. To achieve this, 61 genes, by in rank ordering of betweennees centrality scores, were selected from which 21 genes were shared through normal and cancer GRNs that removed from further investigation. Finally, 40 genes were selected as potential key connectors considered as DCGs specific to cancer samples. Within a network, DCGs, FRZB, SOX9, MMP7, and WDR78 were ordered as the highest strongly connected genes; all up-regulated across different CRC transcriptome experiments in GEMMA database (Fig. 5). Pathway annotation is usually performed by taking overlap of a gene set with a pathway that increases false positives and false negatives[30]. However, PathwAX web server in addition to network crosstalk enrichment can perform depletion analysis. A significant depleted pathway suggests that the links between genes is not much significant to be affected by a certain pathway. Here, DCGs were enriched with ECM-receptor interactionby ITGA2 and TGFB1 and synaptic vesicle cycle by GART, ABCC1, *STMN2*, *EIF5A2* and *SLC7A5*, while cell cycle pathway depleted significantly at *q* value of 0.05 (Fig. 8). ECM- receptor proteins have been dysregulated in the progression from an isolated tumor to metastatic phase[54] and shown to be related to CRC[55]. This is likely emphasizing the role of DCGs in CRC invasion by disseminating the tumor to secondary sites of body. Screening the DCGs across 12 cancer types (bladder, breast, colon, esophagus, kidney, liver, lung, skin, ovary, prostate, sarcoma, and thyroid cancers), suggested that these genes could be speculated as metastatic-related genes as DCGs-shared GART and SLC7A5 with 12 tumor types. The over-representation of DCGs across multiple cancer types with cytokines suggests the explosion of inflammatory events as a similarity among these cancers. DEGs extracted from 12 cancers types shares pathways with DEGs such as cytokines, metabolism, and signaling pathways. Therefore, DCGs likewise are oriented to distinct cascade running the CRC metastasis, while cell cycle is depleted with DCGs. Thus, the specific roles of DCGs in CRC seemto be triggering the metastasis in contrast to their common roles as mediators in TNF, epithelial cell signaling, metabolism, and transportations that was shared with different cancer types.

To evaluate whether the DCGs have any relevance to diseases, we obtained an intersection of DCGs with DisGeNET genes, thereof 11 DGCs (25%) were found to be shared with CRC-related genes in DisGeNET. In fact, this amount of similarity highlights the DCGs with higher ranks in centrality measure, which potentially play remarkable roles in CRC compared to the other genes. However, the low amount of coverage of DCGs with experimentally validated NCG suggests potential unknown genes related to the CRC as targets for future studies. The DCGs were regulated with distinct TFs from which SP1, SF1, and JUN were found to regulate the majority of DCGs. MMP7, SOX9, and CXCL1 found to be regulated by diverse TFs with SP1 as a common regulator (Fig. 6A). This observation may imply that TFs play specific roles in modulating highly interconnected nodes in CRC network. MMP7 and SOX9 are shown to be nodes with the highest connectivity in DCGs (Fig. 4B). MMP family has proven to act in metastatic phase by degrading ECM structures, thus paving the way for the cells through the dense environment[56,57]. Interestingly, SPA1 was the regulator of DCGs implicated in EMC-receptor like ITGA2, ABCC1, and SLC7A5. Integrins, including TGA5, ITGA5, ITGB5, ITGA11, and ITGBL1 elevated in cancer tissues. In accordance with our study, ITGA2 showed up-regulation of 2> fold. Sp1 has been acknowledged to enhance or repress gene expression that in turn plays pivotal roles in metastasis of various tumors[58,59]. Keeping with this analysis, SP1 is likely implicated in CRC progression by regulating EMC components like SLC7A5. It has also been recovered as mutual differentially expressed in 12 screened cancers.

We aimed to delineate prognostic biomarkers underlying CRC; therefore, in the frame of *in silico* analysis, a certain number of genes were explored whose reciprocal interplays are supposedly associated with CRC. Taken together, interactions of these genes which majorly occurred through the metastatic cascade could be considered as the mediators of CRC aggression. Indeed, the identifying these genes exhibits the importance of network topology analysis to rank more important genes as disease-related biomarkers against a set of exclusively DEGs in a meaningful way. These ranks measure the relative importance of a protein in a biological network and could identify strongly correlated genes with specific states. However, this analysis is challenged by the disadvantage of inevitable overestimation in computational approaches; thus, applying more stringent parameters in predicting the regulatory links would be apparently helpful in acquiring more reliable results and overcoming any inaccuracy coming from the nature of reverse engineering methods. Moreover, we employed unweighted networks during GRN reconstruction and topology analysis. We then should be cautious about dynamic nature of cancers via strictly analysis of statics networks.

The main goal of this analysis was exploiting differential connectivity that is thought to rank the influential genes in the pathogenesis of CRC. Utilizing gene expression data with pooling information of TFs in cancers can help to discover crucial findings to identify underlying mechanisms and enlighten more molecular underpinnings of different cancers. We observed that the identified genes and TFs are mainly guided to cytokine signaling pathway and metabolism implicated in CRC. To summarize, selected genes viz. *GART*, *TGFB1*, *ITGA2*, *SLC16A5*, *SOX9*, and *MMP7* with differential connectivity across normal and CRC samples along with SP1, SF1, and JUN TFs could be taken into account for future detection and therapeutic targets by experimental investments.
